# Effects of Phenolic Acids on the Growth and Production of T-2 and HT-2 Toxins by *Fusarium langsethiae* and *F. sporotrichioides*

**DOI:** 10.3390/molecules21040449

**Published:** 2016-04-04

**Authors:** Elena Ferruz, Vessela Atanasova-Pénichon, Marie-Noëlle Bonnin-Verdal, Gisèle Marchegay, Laëtitia Pinson-Gadais, Christine Ducos, Susana Lorán, Agustín Ariño, Christian Barreau, Florence Richard-Forget

**Affiliations:** 1 Veterinary Faculty, Instituto Agroalimentario de Aragón (IA2), Universidad de Zaragoza-CITA, c/Miguel Servet 177, 50013 Zaragoza, Spain; eferruz@unizar.es (E.F.); sloran@unizar.es (S.L.); 2 MycSA, INRA, 71 Avenue Edouard Bourlaux, CS 20032, 33882 Villenave d´Ornon cedex, France; vatanaso@bordeaux.inra.fr (V.A.-P.); Marie-Noelle.Verdal@bordeaux.inra.fr (M.-N.B.-V.); Gisele.Marchegay@bordeaux.inra.fr (G.M.); lpinson@bordeaux.inra.fr (L.P.-G.); christine.ducos@bordeaux.inra.fr (C.D.); cbarreau@bordeaux.inra.fr (C.B.); fforget@bordeaux.inra.fr (F.R.-F.)

**Keywords:** *Fusarium langsethiae*, *Fusarium sporotrichioides*, type A trichothecene, phenolic acid, *Tri* gene

## Abstract

The effect of natural phenolic acids was tested on the growth and production of T-2 and HT-2 toxins by *Fusarium langsethiae* and *F. sporotrichioides*, on Mycotoxin Synthetic medium. Plates treated with 0.5 mM of each phenolic acid (caffeic, chlorogenic, ferulic and *p*-coumaric) and controls without phenolic acid were incubated for 14 days at 25 °C. Fungal biomass of *F. langsethiae* and *F. sporotrichioides* was not reduced by the phenolic acids. However, biosynthesis of T-2 toxin by *F. langsethiae* was significantly reduced by chlorogenic (23.1%) and ferulic (26.5%) acids. Production of T-2 by *F. sporotrichioides* also decreased with ferulic acid by 23% (*p* < 0.05). In contrast, *p*-coumaric acid significantly stimulated the production of T-2 and HT-2 toxins for both strains. A kinetic study of *F. langsethiae* with 1 mM ferulic acid showed a significant decrease in fungal biomass, whereas T-2 production increased after 10 days of incubation. The study of gene expression in ferulic supplemented cultures of *F. langsethiae* revealed a significant inhibition for *Tri5*, *Tri6* and *Tri12* genes, while for *Tri16* the decrease in gene expression was not statistically significant. Overall, results indicated that phenolic acids had a variable effect on fungal growth and mycotoxin production, depending on the strain and the concentration and type of phenolic acid assayed.

## 1. Introduction

T-2 toxin (T-2) and HT-2 toxin (HT-2) are two closely related type A trichothecenes produced by several *Fusarium* species, mainly *F. langsethiae* and *F. sporotrichioides* [1,2]. These mycotoxins may contaminate harvested grain and feed and food products derived thereof. Their presence in cereals such as oat, barley, maize and wheat in cold temperate climates of northern Europe is well documented [3,4,5,6]. T-2 and HT-2 are commonly found together in cereals, as HT-2 is a diacetylated form of T-2 [4,7]. T-2 toxin and HT-2 toxin are toxic to all animal species, as well as to humans. The toxic effects of T-2 and HT-2 include the inhibition of protein synthesis and hematopoiesis, lymphoid depletion and necrotic lesions. The consumption of moldy grains with high levels of T-2 has been associated with human intoxications, such as the Alimentary Toxic Aleukia (ATA), producing sepsis, hemorrhages and pancytopenia [8,9].

In view of their high toxicity and known presence in food and feed, T-2 and HT-2 toxins are considered a potential risk for animal and human health. Thus, a Tolerable Daily Intake (TDI) of 100 ng/kg body weight for the sum of T-2 and HT-2 has been established, which is the lowest TDI of all *Fusarium* toxins.

Conversely, even though maximum limits of T-2 and HT-2 in food products are not yet established due to the limited information available on their occurrence, the European Commission (EC) has recently published indicative values for the sum of T-2 and HT-2 in cereals and cereal products above which investigations should be performed [10]. The recommended levels specified for cereal grains and products for human consumption range from 15 µg/kg in cereal-based foods for infants and young children to 200 µg/kg in oat, oat bran and flaked oats.

Once they have been synthesized, mycotoxins are very difficult to remove, therefore control and prevention strategies in the field and during storage are important to minimize their presence in food and feed. To this end, fungicides have been widely used to control *Fusarium* growth and mycotoxin production both pre and post-harvest. However, legislation concerning the use of these compounds has become tougher in recent years. Besides, most of the synthetic fungicides are frequently perceived to present a hazard to human health and to the environment [11].

Therefore, there have been intensive efforts to find natural alternatives to fungicides that could be used safely to partially or completely inhibit the growth of fungi as well as mycotoxin biosynthesis. Among natural products, the ones which have been the most studied are the phenolic acids, which represent the most common form of phenolic compounds in whole grains and have been reported as *in vitro* inhibitors of *Fusarium* spp. growth [12,13,14,15,16] as well as other fungal genus such as *Aspergillus* spp. [17]. As regards *in vitro* studies on mycotoxin production, phenolic acids showed a significant inhibitory effect on type B trichothecenes [16,18,19,20] and fumonisin B1 [11,14,21].

Real-time PCR has been widely used to evaluate the effects of fungicide treatments on fungal gene expression. The most representative genes associated with type A trichothecene biosynthesis have been described in several studies [22,23]. Trichothecene production is driven by the transcription of the *Tri5* gene which expression is positively regulated by *Tri6* transcriptional regulator. *Tri16* gene is responsible for structural variation at C-8 of type A trichothecenes, while *Tri12* is a transporter gene.

The effect of fungicides [6,24] and of environmental conditions such as water activity and temperature [3,25,26] on fungal growth and T-2 and HT-2 mycotoxin production by *F. langsethiae* was the subject of recent investigations. However, as far as we know, no studies have been performed yet on the effects of phenolic acids on the growth and production of T-2 and HT-2 toxins by *F. langsethiae* and *F. sporotrichioides*, as well as dealing with the effects of phenolic acids at the transcriptional level on the genes involved in type A trichothecene biosynthesis.

A large diversity in phenolic acids content has been found among cultivars of many cereal crops depending on genetic, environmental and agronomic variability. Therefore, breeding cereal varieties with increased content in bioactive phenolic acids could be a useful pre-harvest strategy for the control of toxigenic fungi [27]. However, it is important to determine the potential of phenolic acids on the inhibition of fungal growth and toxin production before extensive breeding work is initiated.

Therefore, the aims of the present study were to determine the *in vitro* effects of four phenolic acids (caffeic, chlorogenic, ferulic and *p*-coumaric), on the fungal biomass and T-2 and HT-2 toxin production by *F. langsethiae* and *F. sporotrichioides.* Additionally, a study of gene expression was carried out on the genes involved in type A trichothecene biosynthesis as affected by ferulic acid.

## 2. Results

### 2.1. Effect of Phenolic Acids on Fungal Growth

Dry fungal biomass of *F. langsethiae* INRA 466 and *F. sporotrichioides* INRA 101 cultivated in Mycotoxin Synthetic (MS) medium supplemented or not with 0.5 mM phenolic acids is reported in Table 1. With the experimental conditions and *Fusarium* strains chosen for the present study, higher biomass values were obtained in the control for *F. sporotrichioides* (28.83 mg) as compared to *F. langsethiae* (19.67 mg). The biomass of both *Fusarium* species was significantly affected by most of the tested phenolic acid treatments at 0.5 mM. Thus, caffeic, chlorogenic and ferulic acids increased fungal biomass of *F. langsethiae* (*p* < 0.05), while the biomass of *F. sporotrichioides* increased with caffeic, ferulic and *p*-coumaric acids (*p* < 0.05).

As regards to the kinetic study of *F. langsethiae*, fungal growth was significantly reduced (*p* < 0.05) with ferulic acid at 1 mM after 6, 10 and 14 days of incubation (Table 2). The higher fungal biomass was achieved after 14 days of incubation, when the control fungal biomass reached 24.7 mg, while that with ferulic acid amounted to 21.1 mg. In addition, in 3-day old broths, only 1% of the added ferulic acid was quantified in liquid media. Ferulic acid was undetectable after 6 days of incubation.

### 2.2. Effect of Phenolic Acids on the Production of T-2 and HT-2 by Fusarium Strains

**Figure 1:**
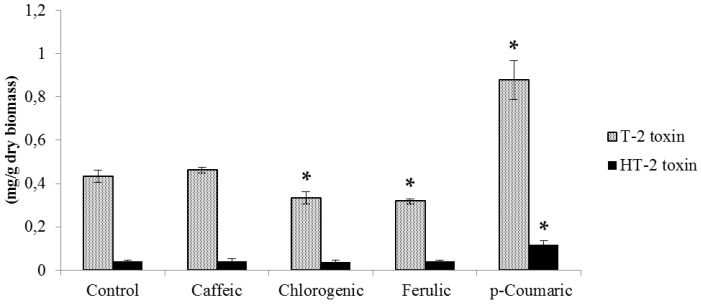
T-2 and HT-2 toxin production by *F. langsethiae* at 0.5 mM of each phenolic acid incubated at 25 °C for 14 days. Values expressed as mean values ± SD of three biological replications. The sign * indicates significant difference with respect to control according to *t*-test (*p* < 0.05).

**Figure 2:**
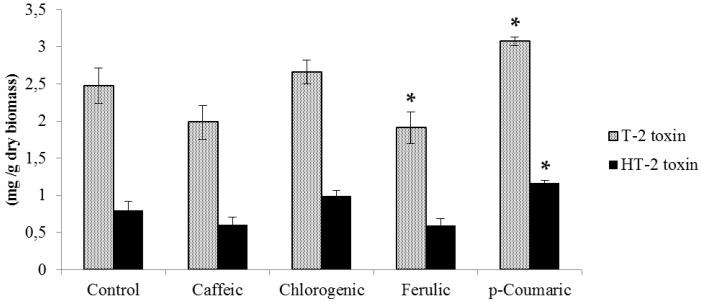
T-2 and HT-2 toxin production by *F. sporotrichioides* at 0.5 mM of each phenolic acid incubated at 25 °C for 14 days. Values expressed as mean values ± SD of three biological replications. The sign * indicates significant difference with respect to control according to *t*-test (*p* < 0.05).

T-2 production was higher than HT-2 in all controls and treatments with phenolic acids in both fungal species under study. Supplementation with 0.5 mM chlorogenic and ferulic acids significantly reduced T-2 biosynthesis by *F. langsethiae* by 23% and 26%, respectively (Figure 1), while HT-2 remained essentially unchanged. Conversely, *p*-coumaric acid increased T-2 and HT-2 production by approximately 2-fold. With regard to toxin production by *F. sporotrichioides* (Figure 2), the tested strain produced up to 2.47 mg/g of T-2 and 0.79 mg/g of HT-2, which is higher than the amount synthetized by the *F. langsethiae* strain. Ferulic acid reduced T-2 production by 23% (*p* < 0.05), while *p*-coumaric acid significantly stimulated the production of T-2 and HT-2 by 24% and 47%, respectively (*p* < 0.05).

**Figure 3:**
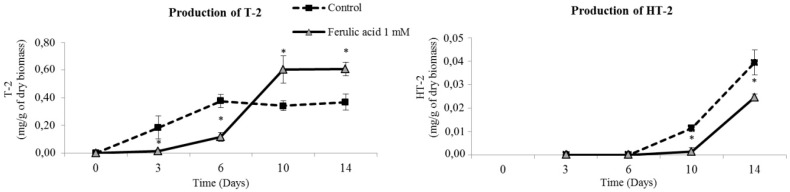
Kinetics of T-2 and HT-2 toxin by *F. langsethiae* INRA 466 at 1 mM of ferulic acid incubated at 25 °C. The sign * indicates significant difference with respect to control according to *t*-test (*p* < 0.05).

In the kinetic study of *F. langsethiae*, T-2 was detected after 3 days of incubation whereas HT-2 biosynthesis started later, between 6 and 10 days of incubation in all plates (both control and treatment with ferulic acid) (Figure 3). After 3 and 6 days of incubation, plates treated with ferulic acid at 1 mM produced significantly less T-2 than controls did (*p* < 0.05). However, after 10 and 14 days of incubation, treated plates were characterized by significantly higher levels of T-2 (0.60 mg/g after 14 days) compared to controls (0.37 mg/g after 14 days) (*p* < 0.05). The contrary effect was observed for HT-2 levels after 10 and 14 days of incubation, which were significantly lower (*p* < 0.05) in ferulic acid plates (0.025 mg/g after 14 days) than in control plates (0.039 mg/g after 14 days). As in the previous *in vitro* study performed with 0.5 mM of phenolic acid, concentration of T-2 was higher than that of HT-2 in all plates.

### 2.3. Effect of Ferulic Acid on Tri Gene Expression by F. langsethiae 

To evaluate effects at the transcriptional level involved in type A trichothecene biosynthesis inhibition by ferulic acid, the expression of several representative genes involved in the biosynthesis pathway (*Tri5*, *Tri6*, *Tri12* and *Tri16*) was analyzed. Expression of all the studied *Tri* genes was lower in ferulic acid supplemented culture as compared with control. A significant inhibition of expression induced by ferulic acid was observed for *Tri5*, *Tri6* and *Tri12* genes, while for *Tri16* gene the decrease was not statistically significant.

**Figure 4:**
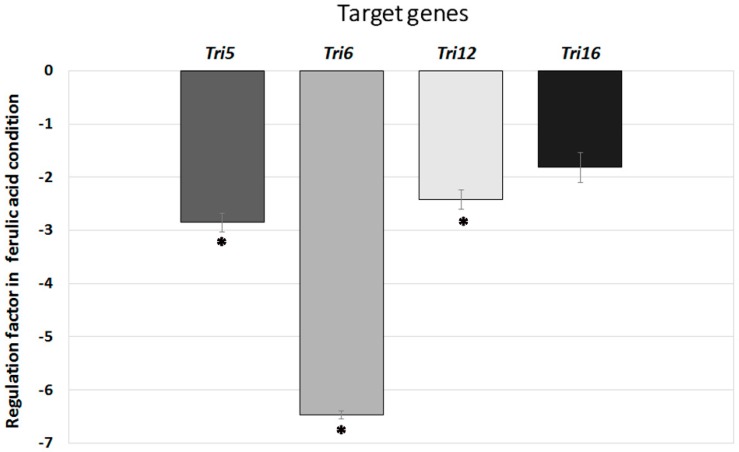
Regulation factors obtained for *Tri* genes in culture supplemented with ferulic acid relative to the control culture. The sign * indicates a significant regulation according to *t*-test (*p* < 0.05).

The regulation factors obtained for the target gene in the ferulic acid supplemented culture relative to the control culture were calculated using the REST software. Thus, the biosynthesis gene *Tri5* was expressed 2.85 times less in ferulic acid treated cultures, while expression of the transcription factor *Tri6* was reduced 6.5 times. Additionally, the expression of the *Tri12* (transporter) and the *Tri16* (C-8 modification) genes were reduced 2.4 and 1.8 times, respectively, in ferulic acid treated cultures. This expression pattern suggests that, in ferulic acid supplemented cultures, the decrease in toxin production could result from a decrease in the level of *Tri* gene expression (see Figure 4).

## 3. Discussion

The present study has evaluated the efficacy of four phenolic acids (caffeic, chlorogenic, ferulic and *p*-coumaric) against *F. sporotrichioides* and *F. langsethiae* in relation to both growth and T-2 and HT-2 production. 

Most phenolic acids tested at 0.5 mM increased the fungal biomass of *F. langsethiae* and *F. sporotrichioides*. However, the effects of phenolic acids on T-2 and HT-2 production were dependent on the type of phenolic acid and the strain. Thus, ferulic acid showed a clear inhibitory effect on T-2 production by *F. langsethiae* and *F. sporotrichioides*, chlorogenic acid only inhibited T-2 synthesis by *F. langsethiae,* while caffeic acid showed little effects on toxin production. Finally, *p*-coumaric acid stimulated T-2 and HT-2 production by both *F. sporotrichioides* and *F. langsethiae*.

As far as we know, there are no available studies on the effects of phenolic acids on the growth of *F. sporotrichioides* and *F. langsethiae* and their impact on T-2 and HT-2 production. However, recent *in vitro* studies have been performed on other *Fusarium* species and mycotoxins. Thus, as observed in the present work, other studies testing the effects of ferulic and *p*-coumaric acids on type B trichothecene production also obtained variable results depending on the strain, type and concentration of phenolic acid. Boutigny *et al.* [19] reported an inhibitory effect of ferulic acid on fungal growth and fully inhibition of type B trichothecene production by *F. culmorum* in MS medium at 2.5 mM. However, lower concentrations (from 0.1 to 1 mM) did not affect fungal growth, while mycotoxin production was highly inhibited, ranging from 68% to 99% inhibition. This result is in accordance with the present study, as 0.5 mM of ferulic acid reduced T-2 synthesis but increased the fungal growth. On the contrary, Picot *et al.* [21] found that the same concentration of ferulic acid did not affect fungal growth or fumonisin production by *F. verticillioides* in Glucose Yeast Extract Peptone (GYEP) medium; though higher concentrations (1 mM) did have an inhibitory effect (53%–86%) on fumonisin biosynthesis.

What’s more, it is worth mentioning that the stimulating effect on mycotoxin production observed with *p*-coumaric acid had been previously described by Ponts *et al.* [16], who reported that GYEP medium supplemented with *p*-coumaric acid at 0.5 mM enhanced type B trichothecene biosynthesis by *F. graminearum*.

In the kinetic study of *F. langsethiae*, ferulic acid at 1 mM reduced fungal growth at all times of incubation. However, while T-2 and HT-2 production were significantly reduced at 3 and 6 days of incubation, this decreasing trend changed after 10 days of culture. At this time of incubation, toxin production was stimulated and concentrations of T-2 and HT-2 in the treated medium were significantly higher (*p* < 0.05) than controls. This shift in the trend could be related to the levels of ferulic acid in the treated plates, which were decreasing from the beginning of the incubation period. Thus, in the third day only 1% of added ferulic acid was still remaining while went undetected on the sixth day. This finding could be hypothetically explained because ferulic acid is being depleted while interfering with fungal metabolism [19]. Once ferulic acid has been fully metabolized, the fungus has no obstacles to produce T-2 and HT-2. Similarly to what was observed with *F. graminearum* and DON production [16], 1 mM ferulic acid can significantly inhibit the breakdown of hydrogen peroxide by the *Fusarium* strain and therefore the enhancement of T2/HT2 accumulation is consistent with the activating effect frequently assigned to hydrogen peroxide on mycotoxin production [28].

Comparing these results with those obtained in the *in vitro* study with ferulic acid at 0.5 mM, it was observed that lower doses stimulated fungal growth and inhibited toxin production, whereas higher doses had the contrary effect. These findings might suggest that sublethal doses of ferulic acid could have an inhibitory effect of toxin production and no effects on fungal growth.

At this concern, Ferrochio *et al.* [11] reported that 1 mM of ferulic acid in maize based medium, stimulated both fungal growth and fumonisin production. However, higher concentrations (20 mM and 25 mM) significantly reduced both *F. verticillioides* growth and fumonisin production. Lower concentrations of 10 mM stimulated fumonisin production while still inhibiting fungal growth.

In relation to the strains assayed, in the present study, *F. langsethiae* was more sensitive to phenolic acids (chlorogenic and ferulic acid reduced toxin production) than *F. sporotrichioides* (only affected with ferulic acid). In addition, *F. sporotrichioides* strain produced higher levels of T-2 and HT-2 than *F. langsethiae*, regardless of the treatment with phenolic acid. This is in accordance with a recent *in vitro* study by Kokonnen *et al.* [1] in which *F. sporotrichioides* strains produced higher concentrations of T-2 and HT-2 than *F. langsethiae* did.

Furthermore, the transcription of the most representative *Tri* genes associated with the type A trichothecene biosynthetic pathway (*Tri5*, *Tri6*, *Tri12* and *Tri16*) was investigated. Results showed a decrease in the relative quantity of *Tri* genes transcription in ferulic supplemented cultures of *F. langsethiae*. This suggests for the first time that inhibition of toxin synthesis by this phenolic acid could be regulated at the transcriptional level. Comparable results have been obtained by Boutigny *et al.* [20] suggesting that natural phenolic acids from wheat bran inhibit *Fusarium culmorum* type B trichothecene biosynthesis *in vitro* by repressing *Tri* gene expression.

Accordingly to the results obtained in this work and in addition to those described by Ferrochio *et al.* [11], it seems that mechanisms of inhibition of *Fusarium* growth by phenolic acids are independent of mechanisms of inhibition behind mycotoxin production. At this concern, our data support the results of Boutigny *et al.* [19] who suggested that type B trichothecenes biosynthesis could be regulated at a transcriptional level, therefore having a regulatory mechanism different from that of fungal growth. Nevertheless, other authors suggested a relationship between growth and mycotoxin production as affected by phenolic compounds [29,30]. All in all, more research is required to elucidate the effects of phenolic acids and the way they regulate fungal growth and mycotoxin synthesis.

## 4. Materials and Methods

### 4.1. Materials

Caffeic, chlorogenic, ferulic and p-coumaric acids were obtained from commercial pure powders purchased from Sigma-Aldrich (Saint-Quentin Fallavier, France). These phenolic acids were selected because of their natural abundance in cereal grains. Standard solutions of T-2 (10 µg/mL) and HT-2 (10 µg/mL) toxins in acetonitrile were obtained from Biopure Standard Solutions (Romer Labs, Tulln, Austria) and stocked at −20 °C until use.

### 4.2. Spore Suspension of Fusarium Strains

*F. langsethiae* INRA 466 and *F. sporotrichioides* INRA 101 were obtained from the INRA-Bordeaux MycSA collection deposited in the International Center for Microbial Resources — Filamentous Fungi (CIRM-CF, http://www6. inra.fr/cirm_eng/Filamentous-Fungi/Strains-catalogue, Marseille, France). Stock cultures were maintained at 4 °C on Potato Dextrose Agar (PDA) (BD Difco™, Illkirch, France) Petri plates. The strains were inoculated and incubated on PDA slants tubes at 25 °C for seven days. The spore suspension was prepared for each strain by adding 6 mL of sterile distilled water to grown agar slants with gentle shaking. The spore suspension was collected directly in another tube and spores were counted on a Thoma cell.

### 4.3. Antifungal Activity Assay

Liquid culture assays were performed in a Mycotoxin Synthetic medium (MS medium) (KH_2_PO_4_, 0.5 g/L; K_2_HPO_4_, 0.6 g/L; MgSO_4_, 0.017 g/L; (NH_4_)_2_SO_4_, 1 g/L; glucose, 20 g/L; biotin, 0.1 mg/L and 0.1 mL/L of Vogel mineral salts solution [31]) as described by Boutigny *et al.* [20]. Phenolic acids were dissolved directly in MS medium to achieve a final concentration of 0.5 mM. An 8 mL aliquot of supplemented MS medium was placed in sterile Petri dishes (55 mm diameter). A control group with no phenolic acid was also prepared. Confirmation of phenolic acid proper supplementation was performed by HPLC- DAD analysis.

Supplemented Petri plates were inoculated with 100 µL spore suspension (10^6^ spores/mL) of *F. langsethiae* and *F. sporotrichioides* and incubated at 25 °C in the dark for 14 days. Cultures were prepared in triplicate. After incubation, cultures were collected in tubes and centrifuged at 3000 g for 10 min in order to separate culture medium from mycelium. Supernatant was stored at −20 °C before quantification of mycotoxins. The mycelium was stored at −80 °C. *Fusarium* spp. biomass was measured by weighing the mycelia after 48 h of lyophilization. Fungal growth was determined as dry weight of mycelium. The pH of culture media was measured before and after incubation in order to check that pH did not change compared to the control.

Following the previous *in vitro* study described above, a kinetic study of T-2 and HT-2 toxin production and fungal growth by *F. langsethiae* INRA 466 was performed to determine the effects of supplementation with 1 mM ferulic acid. Ferulic acid was selected because it is the most abundant phenolic acid in cereal grains. Petri plates (55 mm diameter) containing MS medium supplemented with 1 mM of ferulic acid were inoculated with a spore suspension of *F. langsethiae* (10^6^ spores/mL) and incubated at 25 °C with four different incubation times (3, 6, 10 and 14 days). Cultures were prepared in triplicate. Fungal growth, T-2 and HT-2 toxin production and ferulic acid concentration were determined at these different incubation times.

### 4.4. Extraction of Total RNA, Preparation of cDNA and RT-PCR Analysis

Total RNA was extracted from 3-day-old mycelium from *F. langsethiae* 466 grown in medium with or without 0.5 mM ferulic acid. Four biological replications were prepared for each condition. Fifty milligrams of frozen mycelium were grinded using a TissueLyser II^®^ (QIAGEN, Courtaboeuf, France) for 1 min at 30 Hz before RNA extraction using the RNeasy Plant Mini Kit (QIAGEN), according to the manufacturer’s instructions. Removal of residual DNA using treatment with the DNA-free^TM^ Kit (Ambion by Life Technology SAS, Carlsbad, USA) was performed according to recommendations of the manufacturer. One microgram of total RNA was reverse transcribed using the SuperScript III First-Strand Synthesis System for RT-PCR (Invitrogen, Life Technology SAS), with oligo(dT)20 primers, according to the manufacturer’s instructions. The cDNA obtained were diluted five folds and stored at −20 °C. Gene expression analysis was performed using 1 µL of each cDNA preparation (corresponding to 10 ng of total RNA input in the reverse transcription) in a 10 µL reaction volume, using the QuantiFast SYBR^®^ Green PCR kit (QIAGEN). A LightCycler^®^2.0 system equipped with the LightCycler software 4.0.5 (Roche Diagnostics, Rotkreuz, Switzerland) was used to carry out the reactions. Amplification efficiency (Table 3) was evaluated for each gene with serial dilutions of the pooled cDNA samples and data analysis was performed as previously described by Ponts *et al.* [16]. Expression levels of the studied genes (*Tri5*, *Tri6*, *Tri12* and *Tri16*) were normalized to the expression of the reference genes β-tubulin and citrate synthase (Table 3). Statistical analysis was performed using the REST R-software (Relative Expression Software Tool).

### 4.5. Analysis of T-2 and HT-2 Toxins by HPLC-MS/MS

A representative sample of culture medium was diluted with 50% methanol, shaken with a Vortex mixer and filtered through 0.45 µm, 15 mm diameter RC Phenex™ filter syringe (Phenomenex, Le Pecq, France) before HPLC-MS/MS analysis.

Concentrations of T-2 and HT-2 toxins in liquid cultures were determined using a QTrap 2000 LC/MS/MS system (AB Sciex, Les Ulis, France) equipped with a Turbo Ion Spray ESI source and a 1100 Series HPLC system (Agilent Technology, Massy, France). A Zorbax Eclipse XDB C18 column (150 mm × 2.1 m, 0.5 µm) (Agilent, France) was used; the temperature of the column was maintained at 30 °C. A gradient elution, consisting in 10% (*v*/*v*) methanol in high purity water with 5 mM ammonium acetate (solvent A) and 90% (*v*/*v*) methanol in high purity water with 5 mM ammonium acetate (solvent B) was employed as follows: 10%–70% B in 10 min, 70%–100% B in 2 min, isocratic elution 100% B for 8 min, 100%–10% B for 1 min, and 9 min post-run reconditioning. The flow rate was kept at 300 µL/min and the injection volume was 20 µL.

Quantification was performed on MRM positive mode by monitoring one transition for each analyte: *m/z* 442.2/215.1 for HT-2 and *m/z* 484.2/215.3 for T-2 toxin. The electrospray interface was used at 400 °C with the following settings: auxiliary gas, 60 p.s.i.; ion spray voltage, 4500 V; declustering potential, 36 and 46 V for HT-2 and T-2, entrance potential, 8.5 and 5.5 V for HT-2 and T-2; collision energy, 19 and 25 eV for HT-2 and T-2 respectively.

External calibration was performed to quantify T-2 and HT-2 toxins (25, 50, 100, 250, 500 and 1000 µg/L). Results were converted in µg/g of dry fungal biomass. Data are reported as mean values ± standard deviation (SD) of three biological replicates.

### 4.6. Analysis of Phenolic Acids by HPLC-DAD

Analysis of phenolic acids was performed to confirm the proper supplementation of MS media as well as to determine the evolution of ferulic acid concentration in the kinetic study. Extraction and determination of phenolic acids was performed according to Boutigny *et al.* [20] with some modifications: culture media were diluted with methanol:water (1:1, *v*/*v*) and filtered through a 0.45 µm syringe filter (Phenomenex) before HPLC-DAD analysis. Separation of phenolic acids was achieved on a Kinetex XB-C18 column (150 mm × 4.6 mm, 2.6 µm) (Phenomenex) maintained at 35 °C. The mobile phase consisted of 2% formic acid in water (*v*/*v*) (solvent A) and acetonitrile (solvent B). Phenolic acids were separated by a gradient elution as follows: linear gradient from 5% to 11% B, 0–18 min; isocratic elution 11% B, 18–22 min, linear gradient 11% to 40% B, 22–35 min; linear gradient 40% to 90% B, 35–36 min; isocratic elution 90% B, 36–41 min; linear gradient from 90% to 5% B, 41–43 min and 5% B for 10 min post-run reconditioning. The injection volume was 5 µL. The flow rate was kept at 1 mL/min for a total run time of 43 min. The UV-VIS spectra were recorded from 190 to 500 nm and peak areas were measured at 320 nm for all phenolic acids. Quantification of phenolic acids was performed by using external calibration. Standard solutions of phenolic acids were prepared by dissolving adequate amounts of standards in methanol-water (1:1, *v*/*v*).

### 4.7. Statistical Analysis

Fungal growth and T-2 and HT-2 toxin production of *Fusarium* strains were analyzed statistically with SPSS software v. 20 (IBM Inc., Armonk, NY, USA), performing a t-Student test (*p* < 0.05) (control against treatment with phenolic acids).

## 5. Conclusions

The results obtained in this study suggest that phenolic acids at a dose of 0.5 mM enhanced the fungal biomass of *F. langsethiae* and *F. sporotrichioides*, while the effects on T-2 and HT-2 production were dependent on the type of phenolic acid and the strain. Ferulic acid reduced T-2 production on both fungal species, while the synthesis of T-2 and HT-2 increased with p-coumaric. In the kinetic study, a higher dose of ferulic acid (1 mM) reduced significantly fungal growth of *F. langsethiae*, but mycotoxin synthesis after 14 days of incubation was increased (T-2) or reduced (HT-2). In the study of the transcriptional activity a reduced *Tri* gene expression in ferulic supplemented cultures of *F. langsethiae* was observed. All these observations indicate that mechanisms for fungal growth and mycotoxin production may be independent. This study may help in further understanding the effects of phenolic acids on growth and toxin production by *Fusarium* fungi, as well as the effect on the trichothecene genes and their transcription.

## Figures and Tables

**Table 1:** Effect of phenolic acids at concentration of 0.5 mM on the dry fungal biomass of cultures incubated at 25 °C for 14 days. Values expressed as mean values ± standard deviation (SD) of three biological replications.

Phenolic Acid	Dry Fungal Biomass (mg)	
	*F. langsethiae*	*F. sporotrichioides*
Control	19.67 ± 0.70	28.83 ± 0.26
Caffeic acid	26.03 * ± 1.79	31.97 * ± 0.48
Chlorogenic acid	24.53 * ± 0.55	29.42 ± 0.43
Ferulic acid	24.27 * ± 1.42	33.74 * ± 1.16
*p*-Coumaric acid	19.63 ± 1.82	30.64 * ± 0.87

* Significant difference with respect to control according to *t*-test (*p* < 0.05).

**Table 2:** Effect of ferulic acid (1 mM) on dry fungal biomass of *F. langsethiae* incubated at 25 °C. Values expressed as mean values ± SD of three biological replications.

Time (days)	Dry Fungal Biomass (mg)	
	Control	Ferulic Acid
3	6.92 ± 1.49	5.07 ± 1.68
6	17.10 ± 1.21	14.51 * ± 0.71
10	22.29 ± 0.75	17.83 * ± 1.31
14	24.70 ± 0.49	21.11 * ± 0.65

* Significant difference with respect to control according to *t*-test (*p* < 0.05).

**Table 3:** Studied genes, primers used for qPCR analysis and PCR efficiency.

Gene	Accession No. Genbank	Forward Primer Sequence 5’–3’	Reverse Primer Sequence 5’–3’	PCR Efficiency
Β-tubulin	AF212817	GGTAACCAAATCGGTGCTGCTTTC	GATTGACCGAAAACG AAGTTG	1.97
Citrate synthase	XM_011318922	GGCTCACCGAGTTCAAGAAG	CTTCTCTTGGGCAAAAGTGC	1.97
*Tri5*	AF449792.1	CTATTCCTTGAGATTACAT	CCTTGTAGAATGACATAAGA	2.00
*Tri6*	JXCE01000103.1; locus tag FLAG_12235	CGCTTTCGAATATGGTGGTT	CCTACGGTGGAGCCTACAAA	2.06
*Tri12*	JXCE01000103.1; locus tag FLAG_05858	GGGCTTGCATATCTTGTGGT	TTCGGCCCTATTCGTACAAC	1.88
*Tri16*	HQ594543.1	GGTCTGGTCTAATCTTACA	CACGACATTACCCATATAAG	2.02

## References

[1] Kokkonen M., Medina A., Magan N. (2012). Comparative study of water and temperature relations of growth and T-2/HT-2 toxin production by strains of *Fusarium sporotrichioides* and *Fusarium langsethiae*. World Mycotoxin J..

[2] Nazari L., Pattori E., Terzi V., Morcia C., Rossi V. (2014). Influence of temperature on infection, growth, and mycotoxin production by *Fusarium langsethiae* and *F. sporotrichioides* in durum wheat. Food Microbiol..

[3] Medina A., Magan N. (2010). Comparisons of water activity and temperature impacts on growth of *Fusarium langsethiae* strains from northern Europe on oat-based media. Int. J. Food Microbiol..

[4] Edwards S.G., Imathiu S.M., Ray R.V., Back M., Hare M.C. (2012). Molecular studies to identify the *Fusarium* species responsible for HT-2 and T-2 mycotoxins in UK oats. Int. J. Food Microbiol..

[5] Lindblad M., Gidlund A., Sulyok M., Börjesson T., Krska R., Olsen M., Fredlund E. (2013). Deoxynivalenol and other selected *Fusarium* toxins in Swedish wheat - occurrence and correlation to specific *Fusarium* species. Int. J. Food Microbiol..

[6] Mateo E.V., Valle-Algarra F.M., Mateo R., Jiménez M., Magan N. (2011). Effect of fenpropimorph, prochloraz and tebuconazole on growth and production of T-2 and HT-2 toxins by *Fusarium langsethiae* in oat-based medium. Int. J. Food Microbiol..

[7] De Angelis E., Monaci L., Mackie A., Salt L., Visconti A. (2014). Bioaccessibility of T-2 and HT-2 toxins in mycotoxin contaminated bread models submitted to *in vitro* human digestion. Innov. Food Sci. Emerg..

[8] EFSA (European Food Safety Authority) (2011). Scientific opinion on the risks for animal and public health related to the presence of T-2 and HT-2 toxin in food and feed. EFSA panel on contaminants in the food chain. EFSA J..

[9] Peraica M., Rašić D. (2012). Review. The impact of mycotoxicoses on human history. Arh. Hig. Rada Toksiko..

[B10-molecules-21-00449] 10.Commission Recommendation 2013/165/EU (OJ L91, p12, 03/04/2013) of 27 March 2013 on the Presence of T-2 and HT-2 Toxins in Cereals and Cereal Products.

[11] Ferrochio L., Cendoya E., Farnochi M.C., Massad W., Ramírez M.L. (2013). Evaluation of ability of ferulic acid to control growth and fumonisin production of *Fusarium verticillioides* and *Fusarium proliferatum* on maize based media. Int. J. Food Microbiol..

[12] McKeehen J.D., Busch R.H., Fulcher R.G. (1999). Evaluation of wheat (*Triticum aestivum* L.) phenolic acids during grain development and their contribution to *Fusarium* resistance. J. Agric. Food Chem..

[13] Bily A.C., Reid L.M., Taylor J.H., Johnston D., Malouin C., Burt A.J., Bakan B., Regnault-Roger C., Pauls K.P., Arnason J.T. (2003). Dehydrodimers of ferulic acid in maize grain pericarp and aleurone: resistance factors to *Fusarium graminearum*. Phytopathology.

[14] Samapundo S., De Meulenaer B., Osei-Nimoh D., Lamboni Y., Debevere J., Devlieghere F. (2007). Can phenolic compounds be used for the protection of corn fungal invasion and mycotoxin contamination during storage?. Food Microbiol..

[15] Coma V., Portes E., Gardrat C., Richard-Forget F., Castellan A. (2011). *In vitro* inhibitory effect of tetrahydrocurcuminoids on *Fusarium proliferatum* growth and fumonisin B1 biosynthesis. Food Addit. Contam..

[16] Ponts N., Pinsons-Gadais L., Boutigny A.L., Barreau C., Richard-Forget F. (2011). Cinnamic-derived acids significantly affect *Fusarium graminearum* growth and *in vitro* synthesis of type B trichothecenes. Phytopathology.

[17] Nesci A., Etcheverry M. (2009). Effect of natural maize phytochemicals on *Aspergillus* section *Flavi* sclerotia characteristics under different conditions of growth media and water potential. Fungal Ecol..

[18] Bakan B., Bily A.C., Melcion D., Cahagnier B., Regnault-Roger C., Philogène B.J., Richard-Molard D. (2003). Possible role of plant phenolics in the production of trichothecenes by *Fusarium graminearum* strains on different fractions of maize kernels. J. Agric. Food Chem..

[19] Boutigny A.L., Barreau C., Atanasova-Pénichon V., Verdal-Bonnin M.N., Pinson-Gadais L., Richard-Forget F. (2009). Ferulic acid, and efficient inhibitor of type B trichothecene biosynthesis and *Tri* gene expression in Fusarium liquid cultures. Mycol. Res..

[20] Boutigny A.L., Atanasova-Pénichon V., Benet M., Barreau C., Richard-Forget F. (2010). Natural phenolic acids from wheat bran inhibit *Fusarium culmorum* trichothecene biosynthesis *in vitro* by repressing Tri gene expression. Eur. J. Plant Pathol..

[21] Picot A., Atanasova-Pénichon V., Pons S., Marchegay G., Barreau C., Pinson-Gadais L., Roucolle J., Daveau F., Caron D., Richard-Forget F. (2013). Maize kernel antioxidants and their potential involvement in *Fusarium* ear rot resistance. J. Agric. Food Chem..

[22] Kimura M., Tokai T., Takahashi-Ando N., Ohsato S., Fujimura M. (2007). Molecular and genetic studies of *Fusarium* trichothecene biosynthesis: Pathways, genes, and evolution. Biosci. Biotechnol. Biochem..

[23] McCormick S.P., Stanley A.M., Stover N.A., Alexander N.J. (2011). Trichothecenes: From simple to complex mycotoxins. Toxins.

[24] Mateo E.M., Valle-Algarra F.M., Jiménez M., Magan N. (2013). Impact of three sterol-biosynthesis inhibitors on growth of *Fusarium langsethiae* and on T-2 and HT-2 toxin production in oat grain under different ecological conditions. Food Control.

[25] Medina A., Magan N. (2011). Temperature and water activity effects on production of T-2 and HT-2 by *Fusarium langsethiae* strains from north European countries. Food Microbiol..

[26] Mylona K., Magan N. (2011). Fusarium langsethiae: Storage environment influences dry matter losses and T-2 and HT-2 toxin contamination of oats. J. Stored Prod. Res..

[27] Kaushik P., Andújar I., Vilanova S., Plazas M., Gramazio P., Herraiz F.J., Brar N.S., Prohens J. (2015). Breeding Vegetables with Increased Content in Bioactive Phenolic Acids. Molecules.

[28] Montibus M., Pinson-Gadais L., Richard-Forget F., Barreau C., Ponts N. (2015). Coupling of transcriptional response to oxidative stress and secondary metabolism regulation in filamentous fungi. Crit. Rev. Microbiol..

[29] Torres A., Ramírez M.L., Arroyo M., Chulze S., Magan N. (2003). Potential for control of growth and fumonisin production by *Fusarium verticillioides* and *F. proliferatum* on irradiated maize grain using anti-oxidants. Int. J. Food Microbiol..

[30] Dambolena J.S., Zygadlo J.A., Rubinstein H.R. (2011). Antifumonisin activity of natural phenolic compounds. A structure- property-activity relationship study. Int. J. Food Microbiol..

[31] Vogel H.J. (1956). A convenient growth medium for *Neurospora* (Medium N). Microb. Gen. Bull..

